# 804. A New Paradigm in Wound Healing: Topical Microdosing Approaches

**DOI:** 10.1093/jbcr/irag033.182

**Published:** 2026-04-06

**Authors:** Manuel Prevedel, Maximilian Moshammer, Simon Schwingenschuh, Ana Villa-Garcia, Martina Carnieletto, Sieglinde Purgstaller, Anita Eberl, Lars-Peter Kamolz, Petra Kotzbeck, Elisabeth Hofmann

**Affiliations:** COREMED – Center for Regenerative and Precision Medicine, JOANNEUM RESEARCH Forschungsgesellschaft mbH, Graz, Austria, Graz, Steiermark; COREMED – Center for Regenerative and Precision Medicine, JOANNEUM RESEARCH Forschungsgesellschaft mbH, Graz, Austria, Graz, Steiermark; HEALTH-Institute for Biomedicine and Health Sciences, JOANNEUM RESEARCH Forschungsgesellschaft mbH, Graz, Austria, Graz, Steiermark; COREMED – Center for Regenerative and Precision Medicine, JOANNEUM RESEARCH Forschungsgesellschaft mbH, Graz, Austria, Graz, Steiermark; Division of Plastic, Aesthetic and Reconstructive Surgery, Department of Surgery, Medical University of Graz, Austria, Graz, Steiermark; HEALTH-Institute for Biomedicine and Health Sciences, JOANNEUM RESEARCH Forschungsgesellschaft mbH, Graz, Austria, Graz, Steiermark; HEALTH-Institute for Biomedicine and Health Sciences, JOANNEUM RESEARCH Forschungsgesellschaft mbH, Graz, Austria, Graz, Steiermark; Division of Plastic, Aesthetic and Reconstructive Surgery, Department of Surgery, Medical University of Graz, Austria, Graz, Steiermark; COREMED – Center for Regenerative and Precision Medicine, JOANNEUM RESEARCH Forschungsgesellschaft mbH, Graz, Austria, Graz, Steiermark; COREMED – Center for Regenerative and Precision Medicine, JOANNEUM RESEARCH Forschungsgesellschaft mbH, Graz, Austria, Graz, Steiermark

## Abstract

**Introduction:**

Difficult to treat wounds, such as burns and chronic wounds, constitute a continuous challenge. One current trend is towards developing dressings that allow for targeted delivery of active substances. However, some promising substances are hormones and while exerting positive effects on wound healing, systemic distribution may have detrimental effects and are to be avoided. We have tested a microdosing approach to evaluate the local effects of two hormones, i.e., leptin and estrogen, on wound healing progression.

**Methods:**

Open-flow microperfusion (OFM) technology was adapted to load and supply leptin and 17-b-estradiol via bacterial nanocellulose (BNC) dressings to superficial wounds in porcine ex vivo and in vivo models. Sampling dermal interstitial fluid (dISF) and blood allowed to determine pharmacodynamics and pharmacokinetics. Leptin- and 17-b-estradiol-loaded BNC-dressings were applied to acute superficial excision wounds and wound healing progression was observed for 6 days, with blood and tissue sample collection for analysis of substance distribution, and of local effects on wound healing parameters.

**Results:**

Systemic distribution was not detected for neither leptin nor 17-b-estradiol. In dISF, leptin was shown to reach maximum concentrations of 0.5-5 ng/ml after 6 hours with levels decreasing after 12 hours. For 17-b-estradiol, constant levels (0.1-1 ng/ml) were observed from 2 to 24 hours. No differences were observed in wound healing progression; however slight differences were observed in epidermis thickness after 17-b-estradiol treatment as compared to mock treated wounds.

**Conclusions:**

Functionalization and personalisation of nanocellulose dressings using microdosing of wound healing promoting substances is a promising approach for future burn wound and chronic wound treatment.

**Applicability of Research to Practice:**

Personalized dressings that enable controlled, localized microdosing of active compounds could support targeted modulation of wound healing without systemic risks, and are compatible with existing dressing workflows and bedside sampling.

**Funding for the study:**

Federal Ministry of Innovation, Mobility and Infrastructure (BMIMI), Republic of Austria.

